# Clinical target volume and organs at risk segmentation for rectal cancer radiotherapy using the Flex U-Net network

**DOI:** 10.3389/fonc.2023.1172424

**Published:** 2023-05-18

**Authors:** Xue Sha, Hui Wang, Hui Sha, Lu Xie, Qichao Zhou, Wei Zhang, Yong Yin

**Affiliations:** ^1^Department of Radiation Oncology, Shandong Cancer Hospital and Institute, Shandong First Medical University and Shandong Academy of Medical Sciences, Jinan, China; ^2^Department of Radiation Oncology, Qingdao Central Hospital, Qingdao, Shandong, China; ^3^Hunan Cancer Hospital, Xiangya School of Medicine, Central South University, Changsha, Hunan, China; ^4^Manteia Technologies Co., Ltd, Xiamen, Fujian, China

**Keywords:** automatic segmentation, Flex U-Net, rectal cancer, clinical target volume, organs at risk

## Abstract

**Purpose/Objective(s):**

The aim of this study was to improve the accuracy of the clinical target volume (CTV) and organs at risk (OARs) segmentation for rectal cancer preoperative radiotherapy.

**Materials/Methods:**

Computed tomography (CT) scans from 265 rectal cancer patients treated at our institution were collected to train and validate automatic contouring models. The regions of CTV and OARs were delineated by experienced radiologists as the ground truth. We improved the conventional U-Net and proposed Flex U-Net, which used a register model to correct the noise caused by manual annotation, thus refining the performance of the automatic segmentation model. Then, we compared its performance with that of U-Net and V-Net. The Dice similarity coefficient (DSC), Hausdorff distance (HD), and average symmetric surface distance (ASSD) were calculated for quantitative evaluation purposes. With a Wilcoxon signed-rank test, we found that the differences between our method and the baseline were statistically significant (P< 0.05).

**Results:**

Our proposed framework achieved DSC values of 0.817 ± 0.071, 0.930 ± 0.076, 0.927 ± 0.03, and 0.925 ± 0.03 for CTV, the bladder, Femur head-L and Femur head-R, respectively. Conversely, the baseline results were 0.803 ± 0.082, 0.917 ± 0.105, 0.923 ± 0.03 and 0.917 ± 0.03, respectively.

**Conclusion:**

In conclusion, our proposed Flex U-Net can enable satisfactory CTV and OAR segmentation for rectal cancer and yield superior performance compared to conventional methods. This method provides an automatic, fast and consistent solution for CTV and OAR segmentation and exhibits potential to be widely applied for radiation therapy planning for a variety of cancers.

## Introduction

Currently, rectal cancer is one of the deadliest malignancies, ranking third in the incidence of malignant tumors and fourth in the mortality rate (1). Chemoradiotherapy (CCRT) followed by surgical resection is typically considered the standard treatment for reducing the incidence of local recurrence for locally advanced rectal cancer (2). Intensity-modulated radiation therapy (IMRT) and volumetric modulated arc therapy (VMAT) have become state-of-the-art methods in current radiotherapy practice, because of their ability to facilitate conformity in the desired target dose and sufficiently spare critical structures (3, 4). However, such precise treatments require that CTV and OARs be accurately delineated, as introduced by the International Commission on Radiation Units and Measurement (ICRU) and the subsequently revised guidelines.

It is a very significant yet time-consuming process to manually draw the contours of tumor targets and organs at risk (OARs) in a slice-by-slice manner on planning CT scans in radiation oncology (5, 6). The segmentation task is performed by radiation oncologists, which need to be rich in knowledge, experience and time to achieve a clinical acceptable quality. Moreover, a large amount of image reading work puts a serious burden on radiologists, and the final decisions may be affected by the inter- and intraobserver segmentation variability (7). It is worth noting that multiple studies have shown that the contouring consensus among different oncologists is poor, which has hindered the ability to systematically assess the quality of radiation therapy plans and is considered a major source of uncertainty (8, 9). Therefore, there is an urgent clinical need for an algorithm that can accurately and automatically segment target volumes and OARs.

During the last few decades, the “Atlas-based autosegmentation (ABAS)” method has been one of the most widespread and successful image segmentation techniques in oncology (10). However, ABAS has two main challenges. First, it is hard to establish a “universal atlas” for every organ due to inconsistencies between the shapes and sizes of the organs in the human body. Moreover, a major disadvantage of employing ABAS is that the deformable registration process is time-consuming. It often must align the target image with multiple atlases, which repeats the registration process several times. Recently, various semi-automatic and automatic segmentation methods for CTV delineation based on manual features and machine learning methods have been developed and validated (11). However, due to the potentially conflicting requirements between images, it is difficult to establish a robust, direct, and objective cost function for graph-based methods.

Recently, emerging deep learning (DL) technologies have achieved considerable advancements in medical imaging segmentation, with U-Net being the most popular algorithm. The widespread used U-Net demonstrates the advantages in terms of accurate segmentation due to its U-shaped structure combined with a fast training speed, context information and a small quantity of data used (12). It is inevitable to innovate and improve U-Net-based approaches owing to the current challenges faced by medical image segmentation. The most important point is that the existence of noise reduces the validity of U-Net in supervised learning, which affects the resulting model performance.

In this work, we proposed a new type of convolutional neural network (Flex U-Net) to automatically segment CTVs and OARs in planning CT scans for rectal cancer. Based on popular U-Net architectures, different from conventional segmentation models, the proposed method uses a register model to correct the noise caused by manual annotation, which is a source of potential error in radiation therapy treatments. Attempting to decouple the noise in the labels from the ground truths through the register network provides a pure training target for the segmentation model. Experimental results show that the Flex U-Net proposed in this paper achieves better performance than traditional methods; thus can provide an automatic, fast and consistent solution for radiation therapy planning.

## Materials and methods

### Data acquisition

A total of 265 patients with locally advanced rectal cancer who received preoperative CCRT followed by surgical resection from January 2016 to January 2021 in our department were retrospectively enrolled in this study. Simulated contrast CT data were acquired on a Brilliance CT Big Bore (Philips Healthcare, Best, the Netherlands) system. The CT images were reconstructed with matrix size of 512 × 512 and thickness of 3.0 mm. Magnetic resonance images were collected for some patients to assist with target determination. Regions of interest (ROIs) and OARs were manually drawn on each image slice in the planning CT scans using the Eclipse TPS (Varian Medical Systems, Palo Alto, CA, USA) or Pinnacle TPS (Philips Radiation Oncology Systems, Fitchburg, WI, USA). Clinical Target Volume (CTV) and Organs at Risk (OARs) delineation was based on the guideline published by international experts (13). A set of outlines of the CTVs and OARs on each patient’s CT image was first manually contoured by a radiation oncologist, then reviewed, edited, and finally approved by responsible another radiation oncologist with more than 10 years of experience.

### Image preprocessing

The deep learning performance of a model can be significantly influenced by intensity variations within the utilized dataset. For instance, different CT scanners may use different reconstruction protocols, different slice thicknesses and different voxel space for specific clinical considerations. Therefore, intensity normalization and voxel spacing-based resampling were applied to the raw data. The image intensity distribution of each patient was *N* (0, 1) after normalization. The median spacing of each axis across all the images was selected as the target spacing. All image preprocessing was performed before training started.

### Flex U-Net network architecture

**Figure 1:**
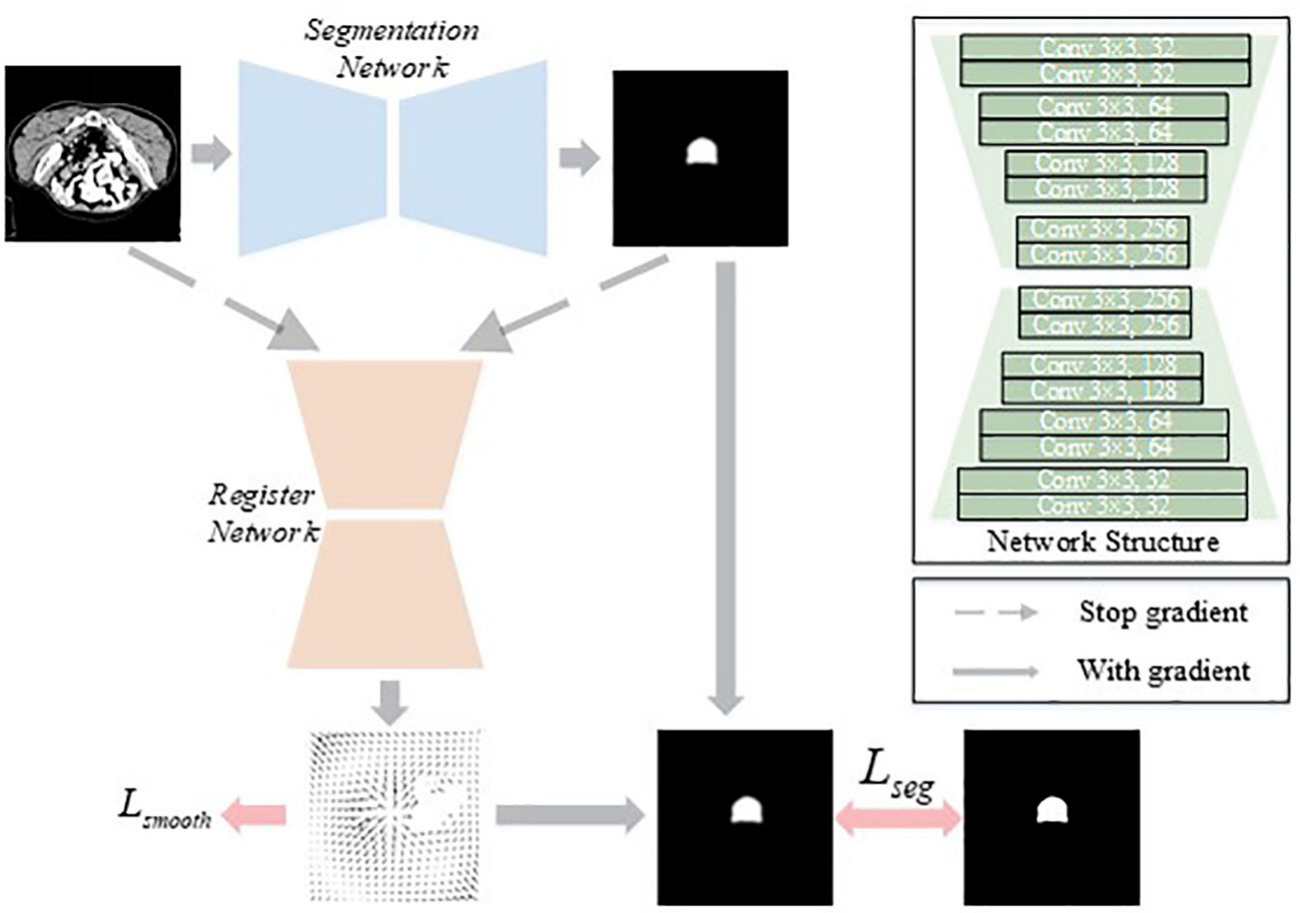
Network architecture of the proposed Flex-UNet.

The proposed Flex U-Net strategy contains two networks: a segmentation network and a register network. As illustrated in **Figure 1**, each image was used as input for the segmentation network to obtain a probability map. This map (gradient stopped) was then fed into the registration network together with the image to obtain a deformation field. The deformation field was then applied to the probability map to obtain a corrected map, which was finally used to calculate the segmentation loss relative to the ground truth.

The theoretical motivation for Flex U-Net stems from the observation of the intra- or interobserver variability that is implicit in labels. There is an inevitable variance caused by the objective determination of the labels during the contouring process. This variance is reflected in the fact that the contour is not sufficient for fitting the real target edge in the given image. Although variance exists in the labels of the dataset, we believe that the overall dataset distribution should be unbiased. Therefore, we decomposed the image segmentation problem into two parts, segmenting the real target edge and performing variance correction, which corresponded to the segmentation network and the register network, respectively.

### Loss function

Suppose that we are given a training dataset $\left\{\left(x_{n},{\hat{y}}_{n}\right)\right\}$ , where $x_{n},{\hat{y}}_{n}$ denote scanned images and manual labels, respectively. ${\hat{y}}_{n}=y_{n}+\epsilon$ indicates that ${\hat{y}}_{n}$ is the real ground truth *y_n_* with a variance of ϵ. Our goal is to train a segmentation network *θ_s_* that can predict the real *y_n_* from the input image *x_n_*, which requires a register network *θ_R_* to correct ${\hat{y}}_{n}$ to *y_n_*. Given the segmentation network probability map $y_{n}^{'}=\theta_{s}\left(x_{n}\right)$ and the deformation field $\lambda_{n}=\theta_{R}(x_{n},y_{n}^{'})$ , the main objective can be expressed as the following equation:


$$L_{M}\left(x_{n},{\hat{y}}_{n}\right)=argmin_{\theta_{R},\theta_{S}}L_{seg}\left(\lambda_{n,}y_{n}^{'},{\hat{y}}_{n}\right)$$

where $\;\lambda_{n},y_{n}^{'}$ denotes the segmentation probability map applied by the deformation field. Specifically, *L_seg_* can be expressed as a combination of the cross-entropy and Dice losses as follows:


$$\begin{array}{l} L_{seg}\left(y^{\prime },y\right)=L_{CE}\left(y^{\prime },y\right)+\alpha \cdot L_{DICE}\left(y^{\prime },y\right)\\ =-\left[y\cdot log\left(y^{\prime }\right)+\left(1-y\right)\cdot log\left(1-y^{\prime }\right)\right]+\alpha \cdot \left(1-\frac{2\cdot \left|y^{\prime }\cap^{}y\right|}{\left|y^{\prime }\right|+\left|y\right|}\right) \end{array}$$

Where *a* is 1.0 by default. To balance the deformation intensity of the register network, the smooth regularization process of the deformation field is introduced as follows:


$$L_{CE}\left(x_{n},y_{n}^{'}\right)=argmin_{\theta_{R}}L_{smooth}\left(\lambda_{n}\right)$$

In summary, our final objective is


$$L\left(x_{n},{\hat{y}}_{n}\right)=argmin_{\theta_{R},\theta_{S}}L_{M}\left(x_{n},{\hat{y}}_{n}\right)+argmin_{\theta_{R}}L_{R}\left(x_{n},y_{n}^{'}\right)$$

### Training details

The deep learning-based training framework used in this study was PyTorch 1.8 LTS. The network structures of the segmentation and register networks were the same, as shown in **Figure 1**. Batch normalization and the rectified linear unit (ReLU) activation function were applied after each convolution layer except for the last layer. The feature maps output by each stage in the encoder module transferred the semantic information to the decoder by concatenation. Data augmentations, including spatial transformation, Gaussian noise addition, Gaussian blurring, and nonlinear intensity shifting, were applied in turn. Other training details included the adaptive moment estimation (Adam) optimizer with a learning rate of 0.0003 and a poly-decay schedule, a total of 100 epochs and 200 iterations per epoch, and a batch size of 16 samples.

### Quantitative evaluation

To assess whether the Flex U-Net correctly segmented the target area, there are 27 patients used to evaluate the performance of the model. We computed several quantitative measurements, namely, the Dice similarity coefficient (DSC), Hausdorff-95 distance (95% HD), and average symmetric surface distance (ASSD), to compare the segmentation results of the proposed method with those of U-Net and V-Net.

The DSC is defined as follows.


(1) $$DSC(A,B)=\frac{2|A\cap B|}{\left|A|+|B\right|}$$

where A represents the ground truth, B denotes the model prediction results and A∩B is the intersection of A and B. The result of DSC is the value ranging from 0 and 1, where 0 reflects no overlap and 1 means there is a complete overlap between structures A and B.

The HD is defined as shown in Eq. (2):


(2) $$HD\left(A,B\right)=max(max_{a\in B}\left(min_{b\in B}d\left(a,b\right)\right),max_{b\in B}\left(min_{a\in A}d\left(b,a\right)\right)$$

where d(a, b) is the distance between point a and point b.

The ASSD is defined as shown in Eq. (3):


(3) $$ASSD=\frac{1}{S\left(A\right)+S\left(B\right)}\left(\sum_{s_{A}\in S\left(A\right)}d\left(s_{A},S\left(B\right)\right)+\sum_{s_{B}\in S\left(B\right)}d\left(s_{B},S\left(A\right)\right)\right)$$

where S (A) represents the surface voxels in set A, and d (v, S (A)) represents the shortest distance from any voxel to S (A).

## Results

The network performance on the independent test group measured by the DSC, HD95 and ASSD metrics is summarized in **Table 1**. The proposed Flex U-Net showed better overall agreement than U-Net and V-Net, as shown by the value of DSC. Compared with U-Net and Flex U-Net, the DSC of CTV_Flex U-Net_ was 0.817, significantly higher than that of CTV_U-Net_ (P = 0.001). Regarding OARs, the differences in DSC of femur head-R (P = 0.001) and smallintestine (P = 0.010) were statistically significant, indicating that Flex U-Net had better segment accuracy. In addition, the HD95 values for all targets were reduced by Flex U-Net compared those of U-Net and V-Net. These values showed the excellent performance of automatic contour segmentation compared with the results of manual segmentation.

**Table 1:** Comparison among the CTV and OAR segmentation results of different models.

		U-Net	V-Net	Flex U-Net	P value
	DSC	0.803 ± 0.082	0.815 ± 0.090	0.817 ± 0.071	0.001
CTV	HD95(mm)	30.0 ± 17.6	16.1 ± 10.5	16.9 ± 11.9	0.057
	ASSD	4.3 ± 2.1	3.9 ± 2.7	4.0 ± 2.7	<0.001
	DSC	0.917 ± 0.105	0.924 ± 0.101	0.930 ± 0.076	0.020
Bladder	HD95(mm)	4.0 ± 2.1	3.9 ± 2.3	3.5 ± 1.4	0.052
	ASSD	0.8 ± 0.4	0.7 ± 0.3	0.7 ± 0.3	0.006
Femur head-L	DSC	0.923 ± 0.030	0.927 ± 0.030	0.927 ± 0.030	0.064
	HD95(mm)	4.0 ± 2.1	3.9 ± 2.3	3.5 ± 1.4	0.011
	ASSD	0.8 ± 0.4	0.7 ± 0.3	0.7 ± 0.3	0.068
Femur head-R	DSC	0.917 ± 0.030	0.922 ± 0.030	0.925 ± 0.030	0.001
	HD95(mm)	4.3 ± 2.3	4.1 ± 2.4	3.9 ± 2.1	0.015
	ASSD	0.8 ± 0.4	0.7 ± 0.4	0.7 ± 0.3	<0.001
	DSC	0.873 ± 0.075	0.882 ± 0.060	0.895 ± 0.053	0.010
Smallintestine	HD95(mm)	12.0 ± 8.5	8.9 ± 6.3	5.3 ± 3.2	<0.001
	ASSD	3.7 ± 2.2	3.2 ± 1.9	2.5 ± 1.3	0.001

## Discussion

Preoperative (chemo) radiotherapy followed by total mesorectal excision is the current standard of care for patients with locally advanced rectal cancer and has been shown to significantly reduce the risk of locoregional recurrence (14, 15). Consistency of target delineation is a key factor in determining the efficacy of radiotherapy. Caravatta et al. (16) evaluated the overlap accuracy between the CTV delineation results of different radiation oncologists and obtained a DSC of 68%. Lu et al. (17) investigated the interobserver variations in the GTV contouring results obtained for H&N patients and reported a DSC value of only 75%. Automatic segmentation of the CTV and OARs has been proposed as a solution to accelerate the delineation process, which is expected to improve the efficiency and consistency of target delineation.

The DL method does not manually extract and learn the information features of the description pattern but discovers a representation of the information through self-learning and uses hierarchical learning abstraction to efficiently complete high-level tasks (18). In the field of computer vision, image segmentation refers to the process of subdividing a digital image into multiple image regions (sets of pixels) that have definite similarity among the features in each region, and the features of different regions exhibit obvious differences. One of the important aspects is that DL has the ability to relieve radiation oncologists from their labor-intensive workloads and increase the consistency, accuracy, and reproducibility of region of interest (ROI) delineation (19). Several authors have applied DL to target volume definition in head and neck cancer (6, 20), prostate cancer (21), lung cancer (22), brain metastases (23), and breast cancer (24).

Target segmentation is the first and key step toward tumor radiotherapy. In other words, image segmentation is about identifying the set of voxels that make up the ROI, which typically can be achieved by employing deep learning methods to medical imaging. Unlike OARs, a CTV is not a well-defined area with clear boundaries but rather includes tissues with the potential for tumor spread or subclinical diseases that are almost undetectable in planning CT images. CTV segmentation depends largely on the radiation oncologists’ knowledge. More specifically, deep learning can reduce the use of domain expert knowledge in the extract and selection of the most appropriate discriminative features.

U-Net is a DL network that segments critical features, and has become a popular baseline in medical imaging segmentation. Nevertheless, these algorithms still do not meet the requirements in the field of radiation therapy. U-Net can be optimized and adjusted according to the actual application scene, and it still has great potential for improvement in terms of training accuracy, feature enhancement and fusion, the use of small sample training set, application range, training speed optimization and so on. This study introduces the modified and developed U-Net models that are suitable for improving segmentation accuracy.

Unlike conventional segmentation models, the proposed method uses a register model to correct the noise caused by manual annotation, which is a source of potential error in radiation therapy treatments. The existence of noise makes the utilized segmentation model learn invalid or even harmful information in supervised learning, which affects the performance of the model. Through the role of the register network, the noise in the labels and the ground truths are decoupled, providing a cleaner training target for the segmentation model. Attempting to decouple the noise in the label from the ground truth through the register network provides a pure training target for the segmentation model. Stefano et al. (25) evaluated a CNN for the automatic segmentation of rectal cancers in multiparametric MR imaging, and the results showed that the average DSC was 0.69. Song et al. (26) used two CNNs, DeepLabv3+ and ResUNet, to segment CTV, and the experimental results showed that the DSCs were 0.79 vs 0.78. Our network exhibited superior performance, and improved the effect of the segmentation model.

Due to data preparation and implementation limitations, there are still some strive to attain better segmentation ability. First, supplementary image modalities can be added to the proposed Flex U-Net to further improve itsd segmentation certainty. Second, the data conducted in this study was from a single center, and all the subjects had the same image parameters. The performance of this study will need to be compared with the results of more prospective studies to confirm our initial findings on the efficiency and accuracy of the method in order to further optimize its performance.

## Conclusion

In this work, we proposed a CTV and OAR segmentation framework for rectal cancer radiotherapy. This research employs a register model to correct the noise caused by manual annotation to refine the performance of the automatic segmentation model. The proposed Flex U-Net is successfully applied to rectal cancer patients and achieves satisfactory CTV and OAR segmentations. Comparison studies proved that our proposed network can reach better segmentation accuracy than conventional U-Net methods, which show great potential to assist physicians in radiotherapy planning for a variety of cancer patients not limited to rectal cancer.

## Data availability statement

The original contributions presented in the study are included in the article/Supplementary Material. Further inquiries can be directed to the corresponding author.

## Author contributions

Conceptualization, XS and YY; methodology, HW; software, LX; validation, QZ and WZ; resources, XS and HS; data curation, HW; writing—original draft preparation, XS; writing—review and editing, YY; visualization, WZ; supervision, HS; project administration, YY; funding acquisition, XS and YY. All authors contributed to the article and approved the submitted version.
